# Myeloid-Derived Suppressor Cells Hinder the Anti-Cancer Activity of Immune Checkpoint Inhibitors

**DOI:** 10.3389/fimmu.2018.01310

**Published:** 2018-06-11

**Authors:** Rebekka Weber, Viktor Fleming, Xiaoying Hu, Vasyl Nagibin, Christopher Groth, Peter Altevogt, Jochen Utikal, Viktor Umansky

**Affiliations:** ^1^Skin Cancer Unit, German Cancer Research Center (DKFZ), Heidelberg, Germany; ^2^Department of Dermatology, Venereology and Allergology, University Medical Center Mannheim, Ruprecht-Karl University of Heidelberg, Mannheim, Germany; ^3^Faculty of Biosciences, Ruprecht-Karl University of Heidelberg, Heidelberg, Germany

**Keywords:** myeloid-derived suppressor cells, immunosuppression, cancer immunotherapy, immune checkpoint inhibition, combination therapy

## Abstract

Immune checkpoint inhibitors (ICI) used for cancer immunotherapy were shown to boost the existing anti-tumor immune response by preventing the inhibition of T cells by tumor cells. Antibodies targeting two negative immune checkpoint pathways, namely cytotoxic T-lymphocyte-associated protein 4 (CTLA-4), programmed cell death protein 1 (PD-1), and programmed cell death-ligand 1 (PD-L1), have been approved first for patients with melanoma, squamous non-small cell lung cancer (NSCLC), and renal cell carcinoma. Clinical trials are ongoing to verify the efficiency of these antibodies for other cancer types and to evaluate strategies to block other checkpoint molecules. However, a number of patients do not respond to this treatment possibly due to profound immunosuppression, which is mediated partly by myeloid-derived suppressor cells (MDSC). This heterogeneous population of immature myeloid cells can strongly inhibit anti-tumor activities of T and NK cells and stimulate regulatory T cells (Treg), leading to tumor progression. Moreover, MDSC can contribute to patient resistance to immune checkpoint inhibition. Accumulating evidence demonstrates that the frequency and immunosuppressive function of MDSC in cancer patients can be used as a predictive marker for therapy response. This review focuses on the role of MDSC in immune checkpoint inhibition and provides an analysis of combination strategies for MDSC targeting together with ICI to improve their therapeutic efficiency in cancer patients.

## Introduction

Cancer immunotherapy has become a promising approach to treat patients over the past decade (1). Furthermore, new types of cancer immunotherapy that are currently under investigation will impact the treatment of cancer patients in the future. Among immunotherapeutic approaches, immune checkpoint inhibition is very promising. However, other types of immunotherapies such as monoclonal antibodies against tumor-associated antigens, cancer vaccines, cell therapy, and unspecific boosting of the immune system with interleukins (IL), interferons (IFN), or toll-like receptor (TLR) ligands are also used and/or under investigation (2).

Immune checkpoint pathways are important to restrict excessive immune responses (3). However, under cancer conditions, tumor cells can exploit these mechanisms to impair or prevent the tumor-targeted immune response. Signals transmitted to T cells either *via* programmed cell death protein 1 (PD-1) or cytotoxic T-lymphocyte-associated protein 4 (CTLA-4) promote T cell anergy and thereby switch off the immune response. Therefore, blockers of these immune checkpoint molecules have been shown to restore an immune response against cancer and increase patient survival (4, 5).

Ipilimumab (monoclonal antibody against CTLA-4) is used for the therapy of cutaneous melanoma. Nivolumab and pembrolizumab (monoclonal antibodies against PD-1) are approved for the therapy of cutaneous melanoma, non-small cell lung cancer (NSCLC), kidney cancer, bladder cancer, head and neck cancers, and Hodgkin lymphoma. Atezolizumab [monoclonal antibody against programmed cell death-ligand 1 (PD-L1)] is approved for the treatment of NSCLC and bladder cancer and avelumab (monoclonal antibody against PD-L1) is approved for gastric cancer and Merkel cell carcinoma therapy. Despite the fact that these immune checkpoint inhibitors (ICI) have proved to be effective, therapeutic resistance occurs in the majority of patients, leading to tumor progression (5, 6). This occurs due to the immunosuppressive tumor microenvironment represented by several immunosuppressive factors and cells, including myeloid-derived suppressor cells (MDSC) (7–10). Importantly, the efficacy of cancer immunotherapy has been reported to be negatively correlated with an increased MDSC frequency and function (11–15).

Myeloid-derived suppressor cells play a leading role in immunosuppression in various cancer types. Accumulating evidences in recent years have even highlighted them as a major driver of an immunosuppressive tumor microenvironment (7–10, 16). Therefore, MDSC could be a promising target in cancer immunotherapy especially in combination with ICI. In this review, we discuss the phenotypic and functional properties of MDSC as well as strategies for their therapeutic targeting. In particular, we address the role of MDSC in immune checkpoint inhibition and provide an analysis of the combination strategies for MDSC targeting together with ICI to improve their therapeutic efficiency in cancer patients.

## Phenotypic and Functional Properties of MDSC

Myeloid-derived suppressor cells represent a heterogeneous population of myeloid cells that fail to differentiate into granulocytes, macrophages, or dendritic cells (DC) but expand during cancer and chronic infection (17–20). They can strongly suppress the activity of T cells, natural killer (NK) cells, and some myeloid cells such as DC (8). MDSC have been identified to expand and play an important role in various cancer types, for example, in patients with melanoma (15, 21–24), multiple myeloma (25), hepatocellular carcinoma (26), NSCLC (27), renal cell carcinoma (28), breast cancer (29), prostate cancer (30), and colorectal cancer (31).

### MDSC Phenotype

In mice, MDSC were characterized by the expression of CD11b and Gr1. However, the use of these markers is no longer sufficient, since MDSC can be divided into two subpopulations in mice: CD11b^+^Ly6G^−^Ly6C^high^ monocytic MDSC (M-MDSC) and CD11b^+^Ly6G^high^Ly6C^low^ polymorphonuclear MDSC (PMN-MDSC) (32). Human M-MDSC are defined as Lin^−^CD11b^+^CD14^+^CD15^−^HLA-DR^−/low^ and PMN-MDSC as Lin^−^CD11b^+^CD14^−^CD15^+^HLA-DR^−^ or Lin^−^CD11b^+^CD14^−^CD66b^+^ (32, 33). One-third subtype of human MDSC, containing more immature HLA-DR^−^CD33^+^CD15^−^CD14^−^ MDSC, has been recently proposed and was termed early stage MDSC (eMDSC) (32).

### MDSC Expansion and Activation

Myeloid-derived suppressor cells are absent in the circulation under homeostatic conditions, but they can be accumulated under pathological conditions like chronic inflammation and cancer (34–39). The expansion and activation of MDSC are controlled by a complex network of soluble factors like IL-6, granulocyte-macrophage colony stimulating factor (GM-CSF), IL-10, M-CSF, G-CSF, and vascular endothelial growth factor (VEGF) as well as TLR ligands (8, 17, 20, 32, 40). The process of MDSC generation is supposed to be divided into two phases that include MDSC accumulation and activation (8, 18–20, 40). MDSC enrichment is mediated by the blockade of the terminal differentiation of immature myeloid cells into granulocytes, macrophages, and DC due to an alteration of the growth factor composition, where G-CSF, GM-CSF, and VEGF play a major role. MDSC activation is mediated by the long-term secretion of cytokines like IL-6, IL-10, IL-1β, and IFN-γ, as well as TLR ligands, such as damage-associated molecular pattern molecules produced under chronic inflammation (8, 18–20, 40).

The production of immunosuppressive factors is driven *via* the Janus kinase (JAK)/signal transducer and activator of transcription (STAT) and myeloid differentiation primary response 88/nuclear factor “kappa-light-chain-enhancer” of activated B-cells signal transduction cascades in MDSC (17, 40).

### MDSC Function

Activated MDSC produce elevated levels of nitric oxide (NO) *via* inducible nitric oxide synthase (iNOS) and upregulate the expression of arginase-1 (ARG-1), both leading to cell cycle arrest in T cells *via* depletion of the amino acid l-arginine from the tumor microenvironment (41, 42) and to T cell anergy induced by the downregulation of T cell receptor (TCR) ζ-chain expression (16, 43). Moreover, NO and reactive oxygen species produced by MDSC can induce T cell apoptosis or TCR nitrosylation (44, 45). In addition, activated MDSC express high levels of PD-L1 (46, 47) that interacts with PD-1 on T cells and causes their exhaustion (48). MDSC also express elevated levels of indoleamine 2,3-dioxygenase (IDO), an enzyme degrading l-tryptophan into N-formylkynurenine (49). The starvation from the amino acid l-tryptophan can lead to T cell arrest and anergy (50). Furthermore, it has been shown to drive the differentiation of CD4^+^ T cells into immunosuppressive regulatory T cells (Tregs) (51). MDSC can also induce Treg expansion and reduction of the anti-tumor activity of effector T cells *via* the expression of CD40 (52) and the secretion of transforming growth factor-β and IL-10 (53–55). Furthermore, MDSC impair the Fc receptor-mediated functions of NK cells by the production of NO (56).

In addition to their immunosuppressive properties, MDSC can have other tumor promoting effects. In particular, they stimulate tumor angiogenesis by secreting VEGF and basic fibroblast growth factor (57, 58). By secreting matrix metalloproteinases (MMP), especially MMP9, they mediate a lower integrity of the extracellular matrix and the basal membrane, which enables tumor cells to enter the blood stream and form metastasis (59, 60). MDSC were also shown to play an important role in the formation of the pre-metastatic niche, a microenvironment in a secondary organ, facilitating metastasis (61).

## MDSC as a Predictive Marker in Immune Checkpoint Inhibition for Cancer Therapy

Myeloid-derived suppressor cells have been reported to be an important prognostic marker for ICI treatment. Interestingly, MDSC levels could be used to predict therapy response or resistance to ipilimumab treatment in metastatic melanoma patients (62). Clinical responders to ipilimumab therapy showed a significantly lower percentage of Lin^−^CD14^+^HLA-DR^−^ M-MDSC in the peripheral blood as compared to non-responders. This finding suggests the use of circulating M-MDSC frequency as a marker of response, since low frequencies identified patients who could benefit from ipilimumab treatment (62). These data are in agreement with the results from another study, showing that a higher M-MDSC frequency prevented ipilimumab-induced activation and expansion of tumor-specific T cells resulting in the lower clinical response (23). It was shown by three more studies that a lower frequency of circulating MDSC at baseline can be used as a predictive marker for ipilimumab treatment of malignant melanoma patients (14, 15, 63). Moreover, in prostate cancer patients treated with a cancer vaccine in combination with ipilimumab, a lower frequency of circulating MDSC was found to correlate with an increased overall survival of patients (64).

## Strategies for MDSC Therapeutic Targeting to Overcome Resistance to ICI

Due to important role of MDSC in tumor-induced immunosuppression, these cells could be a promising target for a combination therapy with ICI. There are three different approaches to target MDSC, namely the inhibition of (i) MDSC accumulation; (ii) MDSC trafficking; and (iii) MDSC-mediated immunosuppression.

### Reduction of MDSC Frequency

To reduce MDSC frequency, the process of myelopoiesis has to be normalized and MDSC accumulation has to be blocked. Some chemotherapeutics were shown to affect MDSC in tumor-bearing hosts. Using the *RET* transgenic mouse model of malignant melanoma, it was demonstrated that ultra-low non-cytotoxic doses of paclitaxel induced a reduction of MDSC numbers and immunosuppressive activity, resulting in an increased survival of melanoma-bearing mice (65). Furthermore, the treatment of pancreatic cancer patients with gemcitabine led to a reduced number of PMN-MDSC (66). In colorectal cancer patients, the treatment with FOLFOX (folinic acid, 5-fluorouracil, and oxaliplatin) resulted in a reduced immunosuppression and a better clinical outcome that could be attributed to a decrease in MDSC frequency and restored anti-tumor immunity (67).

It has been described that the blockade of retinoic acid signal transduction by all-trans retinoic acid (ATRA) led to the differentiation of MDSC into macrophages and DC in murine and human cell samples (68). ATRA has been applied in two clinical trials, including patients with metastatic renal cell carcinoma and late stage small cell lung cancer, leading to a reduction of MDSC frequencies and an improvement of the patient survival (69, 70).

### Blockade of MDSC Recruitment

To exhibit their immunosuppressive phenotype, MDSC have to be recruited to the tumor site. This process is mediated mainly by chemokines secreted in the tumor microenvironment and chemokine receptors expressed on MDSC (71, 72). The role of C-C motif chemokine ligand (CCL)2 and its receptors C-C chemokine receptor (CCR)2 and 4 in the recruitment of M-MDSC has been well-documented (71, 73). Moreover, it was recently found that CCR5 is expressed on MDSC in *RET* transgenic melanoma-bearing mice and melanoma patients, playing an important role in their recruitment to the tumor microenvironment *via* the CCR5 ligands (CCL3, CCL4, and CCL5) (74, 75). Interestingly, CCR5^+^ MDSC were reported to display higher immunosuppressive potential than their CCR5^−^ counterpart both in mice and patients (74). Moreover, the blockade of the interaction of CCR5 with its ligands by a mCCR5-Ig fusion protein significantly improved the survival of melanoma-bearing animals (74). In addition, in a prostate cancer mouse model, the recruitment of CD11b^+^Gr1^+^ MDSC could be blocked by a CXC chemokine receptor 2 antagonist, thereby potentiating the therapeutic effect of the chemotherapeutic drug docetaxel (76).

### Inhibition of MDSC-Mediated Immunosuppression

Phosphodiesterase-5 inhibitors (sildenafil, tadalafil, and vardenafil) are currently in clinical use for non-tumor conditions (77). However, sildenafil was already shown in several transplantable tumor mouse models to downregulate ARG-1 and iNOS expression in MDSC reducing thereby their immunosuppressive capacity and leading to an enhanced intratumoral T cell infiltration and activation, a reduction of tumor growth, and an improvement of the anti-tumor efficacy of adoptive T cell therapy (78). In the *RET* transgenic melanoma mouse model, sildenafil could also prolong mouse survival that was associated with reduced levels and activity of MDSC in the tumor microenvironment and, therefore, with a restored CD8^+^ T cell infiltration and function (79). Furthermore, in an inflammation-dependent murine colon cancer model, sildenafil prevented tumorigenesis by inhibiting tumor infiltration with MDSC (80).

In clinical trials, tadalafil was applied in patients with head and neck squamous cell carcinoma and metastatic melanoma (81–83). It improved clinical outcome and augmented the anti-tumor immune response of patients due to the reduction of peripheral and tumor-infiltrating MDSC, highlighting thereby its potential application in combined immunotherapy (81–83).

Another promising approach is targeting of STAT3, since it is a main regulator of MDSC immunosuppressive activity (8, 18–20, 40, 84). Systemic administration of the STAT3 antisense oligonucleotide inhibitor AZD9150 was already tested in a phase I clinical trial in patients with lung cancer and lymphoma (85). It has been recently developed a strategy aiming to target STAT3 decoy oligonucleotides specifically to myeloid cells by coupling them to the TLR9 ligand CpG, which led to a reduced ARG-1 expression and to the restoration of T cell functions in patients with acute myeloid leukemia (86).

## Combination of ICI and MDSC Neutralization

**Figure 1:**
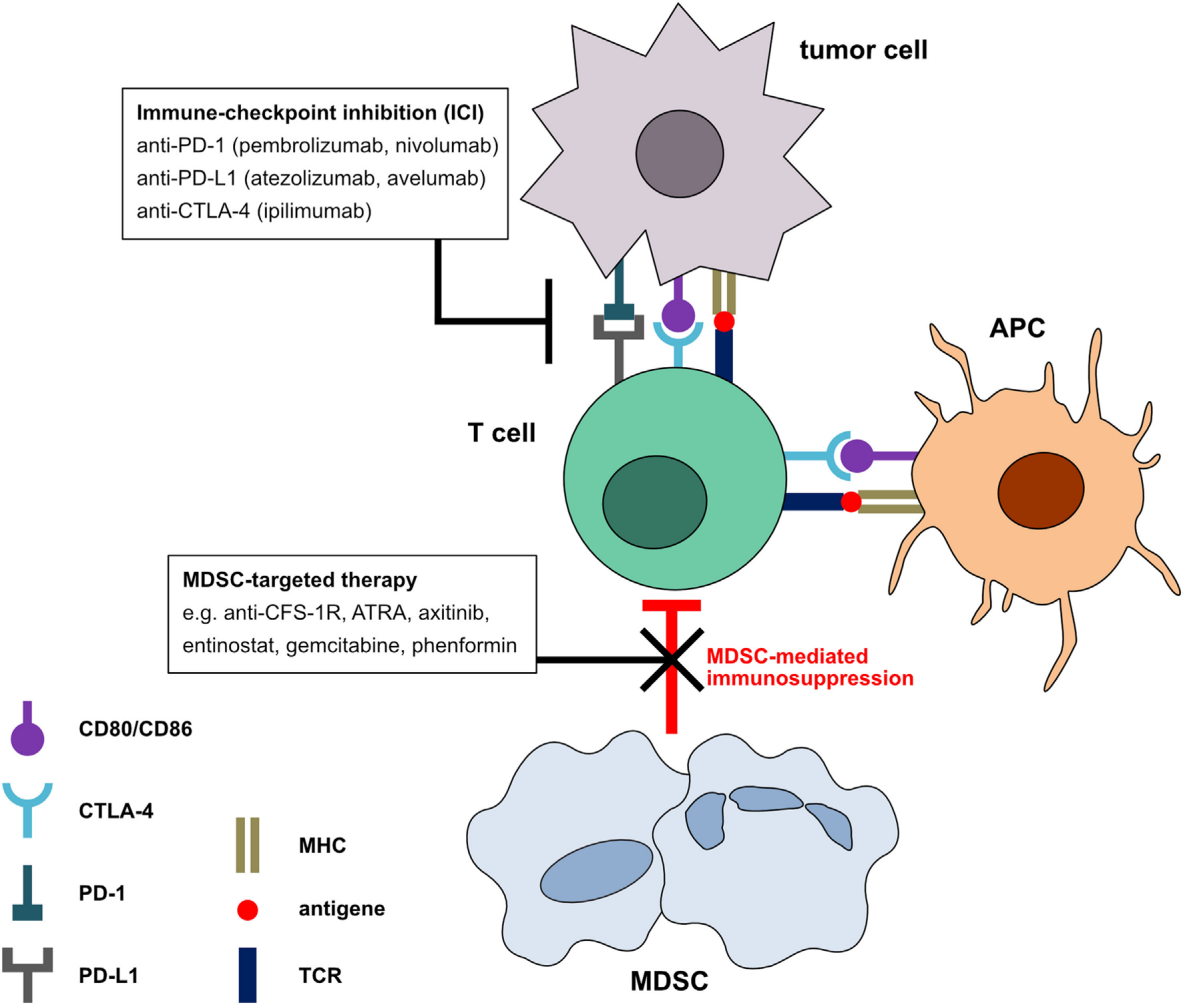
Mechanism of immune checkpoint inhibitors (ICI) in combination with myeloid-derived suppressor cell (MDSC) neutralization. In the tumor microenvironment, tumor and antigen-presenting cells express the ligands for programmed cell death protein 1 and cytotoxic T-lymphocyte-associated protein 4 receptors on T cells. Signals transmitted *via* these receptors induce a T cell arrest and the termination of the anti-tumor immune response. Blockade of these negative checkpoint molecules can restore the anti-tumor activity of T cells. However, MDSC can induce T cell inhibition by mechanisms different from negative checkpoint molecules. The combination of MDSC inhibition with ICI could further increase T cell-mediated anti-tumor immune responses and the clinical outcome of cancer patients.

In recent years, the combination of MDSC targeting with ICI treatment has been applied in preclinical tumor models and cancer patients. Figure 1 illustrates the effect of combination of ICI and MDSC-targeted therapy to enable an anti-tumor immune response. Interestingly, it was shown that anti-PD-1 antibodies themselves seem to have a direct effect on peripheral blood mononuclear cells (PBMC) from cancer patients. It was reported that anti-PD-1 antibodies stimulated *in vitro* PBMC proliferation induced by anti-CD3 antibodies and inhibited the induction of MDSC in the same experimental settings (87).

### ICI Plus Reduction of MDSC Frequency

In two different tumor mouse models, the reduction of MDSC by a histone-deacetylase inhibitor, entinostat, in combination with antibodies against CTLA-4 and PD-1 led to 80% tumor eradication although the application of these ICI alone failed to induce anti-tumor effects (88). In Lewis lung and renal cell carcinoma mouse models, MDSC blocking by entinostat in combination with PD-1 blockade resulted in a significantly increased survival in comparison to anti-PD-1 therapy alone (89). Furthermore, MDSC inhibition by phenformin, an antidiabetic drug from the biguanide class, was able to enhance the effect of PD-1 blockade reflected by an increased CD8^+^ T cell infiltration in the BRAF V600E/PTEN-null melanoma mouse model (90).

In a murine oral cancer model, anti-Ly6G antibodies were applied to deplete PMN-MDSC that resulted in the restoration of antigen-specific T cell responses but failed to improve mouse survival (91). However, the combination of anti-Ly6G and anti-CTLA-4 antibodies induced a complete tumor rejection (91).

### ICI Combined With an Alteration of MDSC Function

In a B16 melanoma mouse model expressing IDO, it has been shown that the blockade of colony stimulating factor 1 receptor (CSF-1R) by the kinase inhibitor PLX647 could inhibit tumor-infiltrating MDSC and enhance anti-tumor T cell responses (92). Moreover, this therapy sensitized the tumor for anti-PD-1 and anti-CTLA-4 antibodies, since the combination therapy led to an increased tumor regression and prolonged mouse survival as compared to the therapy with ICI alone (92). The same effect could be shown in CT26 colon and 4T1 breast cancer mouse models, where the combination of anti-CTLA-4 treatment with CSF-1/CSF-1R blockade enhanced the beneficial effect by reprogramming MDSC (93). Moreover, the expression of CSF-1 on tumor cells in melanoma and NSCLC patients correlated with the enrichment of MDSC that could be inhibited *in vitro* by the blockade of CSF-1/CSF-1R signaling (93). This observation was supported by another study, demonstrating that the blockade of M-CSF/CSF-1R interaction by BLZ945 could result in an improved efficacy of PD-1 blockade by inhibiting MDSC in mice with neuroblastoma (94).

The blockade of the VEGF receptor by axitinib in combination with anti-CTLA-4 antibodies increased survival of mice with subcutaneous melanoma and intracranial melanoma metastasis (95). This effect was due to an increased antigen-presenting capacity of DC and to a reduced suppressive capacity of M-MDSC, inducing the stimulation of CD8^+^ and CD4^+^ T cells (95).

Importantly, ICI treatment of head and neck cancer was reported to be noneffective due to the recruitment of MDSC (96). However, the treatment of mice bearing head and neck tumors with IPI-145, an inhibitor of phosphatidylinositol-4,5-bisphosphate 3-kinase (PI3K)δ and PI3Kγ isoforms, in combination with anti-PD-L1 antibodies resulted in the inhibition of MDSC activity associated with CD8^+^ T cell-dependent delay of tumor growth and with an improved survival (97).

It has been demonstrated that cell cycle-related kinase (CCRK) from human hepatocytes stimulated an expansion of CD11b^+^CD33^+^HLA-DR^−^ MDSC *via* an NFκB/IL-6-dependent mechanism (98). Similarly, in CCRK transgenic mice, PMN-MDSC frequency and activity were shown to be increased. Thus, upon inhibition of CCRK, PMN-MDSC numbers were decreased, an increased infiltration of IFN-γ^+^TNF-α^+^CD8^+^ T cells was observed, and tumor progression was impaired (98). The beneficial effect was even stronger upon the combination with anti-PD-L1 antibodies (98).

### Ongoing Clinical Trials

Some strategies modulating MDSC frequency and immunosuppressive function are already used in various clinical trials in combination with ICI (Table 1). Thus, a combined therapy with the anti-PD-L1 antibody atezolizumab and the histone-deacetylase inhibitor entinostat is currently under investigation in a phase I/II clinical trial in renal cell carcinoma patients. Furthermore, ATRA was applied in combination with ipilimumab in a phase II clinical trial in melanoma patients, inducing an improvement of clinical outcome associated with increased tumor antigen-specific T cell responses and decreased MDSC frequencies as compared to ipilimumab alone (99). Two other clinical trials in melanoma patients are utilizing the combination of ICI treatment with MDSC targeting by SX-682, a small-molecule dual-inhibitor of C-X-C motif chemokine ligand 1 and 2, or by the antioxidative and anti-inflammatory drug omaveloxolone (RTA 408). Since it was shown that gemcitabine induced a reduction in MDSC numbers in pancreatic cancer patients (66), potentially increasing thereby the efficacy of nivolumab treatment, the combination of these drugs is applied in a phase II clinical trial in NSCLC patients. Furthermore, the tumor necrosis factor-related apoptosis inducing ligand (TRAIL) receptor 2 blocking antibodies DS-8273a, targeting MDSC in cancer patients (100), were applied in a phase I clinical trial in colorectal cancer patients in combination with nivolumab.

**Table 1:** Clinical trials combining myeloid-derived suppressor cell (MDSC) targeting with immune checkpoint inhibitors (ICI) in cancer patients.

No	Title	Disease or conditions	Interventions	Trial number
1	Atezolizumab in combination with entinostat and bevacizumab in patients with advanced renal cell carcinoma	Advanced renal cell carcinoma	Atezolizumab, entinostat, bevacizumab	NCT03024437

2	Ipilimumab and all-trans retinoic acid (ATRA) combination treatment of stage IV melanoma	Melanoma	ATRA, ipilimumab	NCT02403778

3	Depletion of MDSC to enhance anti-programmed cell death protein 1 therapy	Non-small cell lung cancer	Nivolumab, gemcitabine	NCT03302247

4	SX-682 treatment in subjects with metastatic melanoma concurrently treated with pembrolizumab	Melanoma	SX-682, pembrolizumab	NCT03161431

5	RTA 408 capsules in patients with melanoma—REVEAL	Melanoma	Omaveloxolone, ipilimumab, nivolumab	NCT02259231

6	Antibody DS-8273a administered in combination with nivolumab in subjects with advanced colorectal cancer	Colorectal neoplasm	DS-8273a, nivolumab	NCT02991196

## Conclusion

Immune checkpoint inhibitors for cancer therapy are approved for the treatment of cutaneous melanoma, NSCLC, kidney cancer, bladder cancer, head and neck cancers, Merkel cell carcinoma, gastric cancer, and Hodgkin lymphoma and could significantly improve the clinical outcome of cancer patients. However, the resistance to ICI after initial response or total lack of response is still a problem. Resistance can be mediated by MDSC, which makes these cells a promising target for combination therapy.

In various preclinical tumor models, it has been reported that MDSC targeting potentiated the effect of ICI and led to a significantly increased survival and even to full tumor regression, which was not observed upon the treatment with ICI alone. However, only six early phase clinical trials are running to date to improve ICI outcome in cancer patients by reducing MDSC-mediated immunosuppression.

Therefore, more combinatorial trials are needed to use the strategies of MDSC neutralization to further improve the outcome of cancer immunotherapy by ICI.

## Author Contributions

RW: writing, review, and revision of the manuscript, preparation and revision of the figure and table. XH: preparation of the figure. VN: preparation of the table. VF, CG, PA, and JU: review and revision of the manuscript. VU: writing, review, and revision of the manuscript and revision of the table and figure.

## Conflict of Interest Statement

The authors declare that the research was conducted in the absence of any commercial or financial relationships that could be construed as a potential conflict of interest.

## Abbreviations

ATRA, all-trans retinoic acid; ARG-1, arginase-1; bFGF, basic fibroblast growth factor; CCR, C-C chemokine receptor; CCL, C-C motif chemokine ligand; CXCL, C-X-C motif chemokine ligand; CCRK, cell cycle-related kinase; CSF-1R, colony stimulating factor 1 receptor; CTLA-4, cytotoxic T-lymphocyte-associated protein 4; DAMP, damage-associated molecular pattern; DC, dendritic cells; GM-CSF, granulocyte-macrophage colony stimulating factor; ICI, immune checkpoint inhibitors; IDO, indoleamine 2,3-dioxygenase; iNOS, inducible nitric oxide synthase; IFN, interferon; IL, interleukin; JAK, Janus kinase; MMP, matrix metalloproteinases; MyD88, myeloid differentiation primary response 88, MDSC, myeloid-derived suppressor cells; NK, natural killer; NO, nitric oxide; NSCLC, non-small cell lung cancer; NF-κB, nuclear factor “kappa-light-chain-enhancer” of activated B-cells; NY-ESO-1, New York esophageal squamous cell carcinoma-1; PBMC, peripheral blood mononuclear cells; PI3K, phosphatidylinositol-4,5-bisphosphate 3-kinase; PDE5, phosphodiesterase-5; PD-1, programmed cell death protein 1, PD-L1, programmed cell death-ligand 1; ROS, reactive oxygen species; Treg, regulatory T cells; STAT, signal transducer and activator of transcription; TCR, T cell receptor; TLR, toll-like receptor; TGF, transforming growth factor; VEGF, vascular endothelial growth factor; VEGFR, VEGF receptor.
